# Setting Research Priorities to Reduce Almost One Million Deaths from Birth Asphyxia by 2015

**DOI:** 10.1371/journal.pmed.1000389

**Published:** 2011-01-11

**Authors:** Joy E. Lawn, Rajiv Bahl, Staffan Bergstrom, Zulfiqar A. Bhutta, Gary L. Darmstadt, Matthew Ellis, Mike English, Jennifer J. Kurinczuk, Anne C. C. Lee, Mario Merialdi, Mohamed Mohamed, David Osrin, Robert Pattinson, Vinod Paul, Siddarth Ramji, Ola D. Saugstad, Lyn Sibley, Nalini Singhal, Steven N. Wall, Dave Woods, John Wyatt, Kit Yee Chan, Igor Rudan

**Affiliations:** 1 Saving Newborn Lives/Save the Children, Cape Town, South Africa; 2 Department for Child and Adolescent Health and Development, World Health Organization, Geneva, Switzerland; 3 Division of Global Health, Karolinska Institutet, Stockholm, Sweden, and Averting Maternal Death and Disability Program, Columbia University, New York, New York, United States of America; 4 Division of Women & Child Health, the Aga Khan University, Karachi, Pakistan; 5 Family Health Division, Global Health Program, Bill & Melinda Gates Foundation, Seattle, Washington, United States of America; 6 Community Child Health Partnership, Southmead Hospital, Bristol, United Kingdom; 7 KEMRI–Wellcome Trust Programme, Centre for Geographic Medicine Research–Coast, Nairobi, Kenya, and Department of Paediatrics, University of Oxford, Oxford, United Kingdom; 8 The National Perinatal Epidemiology Unit, University of Oxford, Oxford, United Kingdom; 9 Department of International Health, Johns Hopkins Bloomberg School of Public Health, Baltimore, Maryland, United States of America; 10 Department of Reproductive Health and Research, World Health Organization, Geneva, Switzerland; 11 George Washington University, Washington, D.C., United States of America; 12 Centre for International Health and Development, UCL Institute of Child Health, London, United Kingdom; 13 MRC Maternal and Infant Heath Care Strategies Research Unit at the University of Pretoria, Pretoria, South Africa; 14 Department of Pediatrics, All India Institute of Medical Sciences, New Delhi, India; 15 Department of Pediatrics, Maulana Azad Medical College, New Delhi, India; 16 Department of Pediatric Research, Oslo University Hospital Rikshospitalet, University of Oslo, Norway; 17 Nell Hodgson Woodruff School of Nursing, Atlanta, Georgia, United States of America; 18 Department of Pediatrics, University of Calgary, Calgary, Alberta, Canada; 19 Saving Newborn Lives/Save the Children, Washington, D.C., United States of America; 20 University of Cape Town and the Perinatal Education Programme, Observatory, South Africa; 21 Centre for Philosophy, Justice and Health, University College of London, London, United Kingdom, and Center for Women's Health, University College of London, London, United Kingdom; 22 Nossal Institute of Global Health, University of Melbourne, Melbourne, Australia; 23 Croatian Centre for Global Health, University of Split Medical School, Split, Croatia, and the Centre for Population Health Sciences, The University of Edinburgh Medical School, Edinburgh, United Kingdom

## Abstract

Joy Lawn and colleagues used a systematic process developed by the Child Health Nutrition Research Initiative (CHNRI) to define and rank research options to reduce mortality from intrapartum-related neonatal deaths (birth asphyxia) by the year 2015.

**Summary Points**

- Intrapartum-related neonatal deaths (previously called “birth asphyxia”) are the fifth most common cause of deaths among children under 5 years of age, accounting for an estimated 814,000 deaths each year, and also associated with significant morbidity, resulting in a burden of 42 million disability adjusted life years (DALYs).
- This paper uses a systematic process developed by the Child Health Nutrition Research Initiative (CHNRI) to define and rank research options to reduce mortality from intrapartum-related neonatal deaths by the year 2015, in order to advance Millennium Development Goal (MDG) 4 for child survival.
- A list of 61 research questions was developed and scored by 21 technical experts. The top one-third of the ranked research investment options was dominated by delivery (implementation) research, whilst discovery (basic science) questions were not ranked highly, especially for expected reduction of mortality and inequity in the short time to 2015.
- Among the top four research questions, two relate to generation of demand for facility care at birth with specific mechanisms (such as transport and communication schemes, or financial incentives and conditional cash transfers). The other two top ranked priorities relate to use of community cadres and the roles they might effectively play—for example, screening for complications or supportive transfer to facilities and companionship at birth. The highest ranked discovery question concerned the interaction of hypoxia and infection, and the highest ranked epidemiologic question addressed prediction of intrapartum hypoxic injury.
- This exercise highlights the need for current research investments to focus on studies most likely to result in accelerated progress towards MDG 4 and in the countries where the most deaths occur.

## Introduction

The Millennium Development Goals (MDGs), ratified by almost every country in the world, have catalyzed policy attention and investment for child survival (MDG 4) and maternal health (MDG 5) [1]. MDG 4 aims for a two-thirds reduction in deaths of children under 5 years of age between 1990 and 2015. Despite almost no progress for MDG 4 on a global level during the 1990s, there has been increasingly rapid progress with several recent landmark achievements since about 2005. The number of child deaths has been reduced to about 8 million per year, despite the continuing increase in the global child population [2],[3], and a number of low-income countries are now on track for the goal [3]. On the African continent, which has had the slowest progress, several countries have moved from the “no progress” to the “rapid progress” group, and two low-income African countries (Eritrea and Malawi) are on track to achieve their MDG 4 goal [4],[5].

## Global Burden of “Birth Asphyxia”

Most of the child mortality reduction in recent decades, however, is attributed to progress in tackling infectious causes of deaths (such as measles, malaria, pneumonia, and diarrhea) in post-neonatal infants and children aged 1–4 years. Reductions in deaths that occur in the neonatal period (the first 28 days after birth) have been relatively limited. When the MDGs were signed in the year 2000, approximately 37% of under-five child deaths occurred in the neonatal period [6]; this has since risen to over 41% [7], a total of 3.6 million deaths. Mortality in the first week after birth, the early neonatal period, has shown the least progress, with no measurable change at global level in the last decade. If progress towards MDG 4 is to be accelerated, then urgent attention is required to reduce neonatal deaths. It also links closely with advancing MDG 5 since women's health and health care, especially at the time of birth, are major determinants of early neonatal deaths, especially those due to preterm birth and complications at birth.

The terms and definitions used to describe a baby affected by birth complications have evolved over time, driven not only by a greater understanding of pathophysiology and clinical manifestations but also by increasing litigation in high-income countries [8]. “Birth asphyxia” is an imprecise term; it was broadly defined by the World Health Organization (WHO) in 1997 as the clinical description of a newborn who “fails to initiate or maintain regular breathing at birth” [9]. This term applies to an important clinical condition—the need for resuscitation—but is not predictive of outcome. Nor does it imply a particular causation (e.g., intrapartum hypoxia) since the baby may not be breathing for other reasons, such as prematurity. Three consensus statements have recommended that terms such as “birth asphyxia”, “perinatal asphyxia”, “fetal distress”, “hypoxic-ischemic encephalopathy”, or “post-asphyxial encephalopathy” should not be used unless evidence of acute intrapartum causation is available [10]–[12]. These consensus statements suggested the term “neonatal deaths associated with acute intrapartum events,” which is cumbersome. Since the late 1990s, the Scottish and UK Confidential Enquiries have included the term “intrapartum-related neonatal death,” which has also been used in a recent supplement on the topic [8]. The terminology used in international health estimates and policy has been slower to change, but in this paper we use the new term, intrapartum-related neonatal deaths.

**Figure 1:**
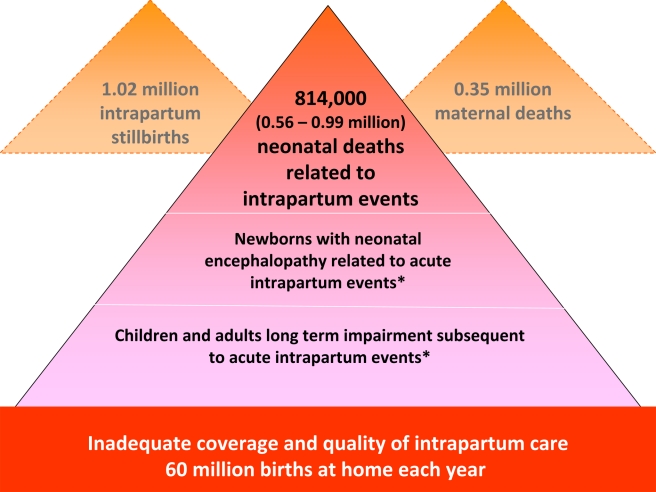
The burden of intrapartum-related neonatal deaths, intrapartum stillbirths, maternal deaths, and the unknown associated burden of neonatal morbidity and disability. Data sources: neonatal deaths [13], stillbirths [15],[16], maternal deaths [48], place of birth [8]. No systematic estimates are currently available.

Each year an estimated 814,000 children die of intrapartum-related causes [13]. Intrapartum-related neonatal deaths are the fifth most common cause of under-five child deaths after pneumonia, diarrhea, preterm birth complications, and neonatal infections [13]. They rarely feature on lists of child survival priorities, compared to other conditions such as malaria that account for fewer child deaths [14]. The burden of intrapartum complications is underestimated if only liveborn babies are considered since an additional 1.02 million stillbirths occur in the intrapartum period [15], which accounts for approximately one-third of the world's total 3.2 million stillbirths [16]. However, stillbirths are not included in MDG tracking or Global Burden estimation (Figure 1). The Global Burden of Disease 2004 report allocated 42 million disability adjusted life years (DALYs) to “birth asphyxia”, which is twice the number of DALYs allocated to diabetes and around 75% of the DALYs for HIV/AIDS [17].

## Mismatch of Burden and Research Investment

**Figure 2:**
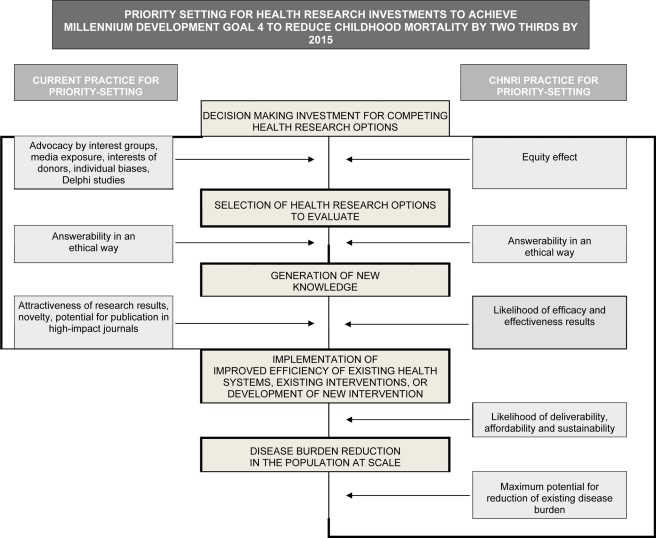
Conceptual framework for Child Health and Nutrition Initiative (CHNRI) showing steps from health research investment to a decrease in burden of death, disease, or disability. Investment decisions in health research are based on a range of factors and processes (left side). The CHNRI framework identifies criteria to discriminate between competing research options (right side): (1) answerability; (2) effectiveness; (3) deliverability; (4) maximum potential for disease burden reduction; and (5) predicted equity effect in the population. These five criteria are used to score the list of research options in the CHNRI research priority setting process [32]–[34].

In evidence-based decision making, research investment would be matched with burden. There is, however, a well-described mismatch between burden and research investment, particularly for conditions common in low-income settings [18],[19]. This mismatch is referred to as the 10/90 gap, whereby 10% of research expenditure is directed at 90% of the world's burden of ill health. The roots of this disparity are complex (see Figure 2, left side). Even in high income countries, the research investment for neonatal deaths is a small fraction of regular investments in research on other conditions [20]. Although the United States National Institutes of Health (NIH) invests approximately US$700 million on research relevant to perinatal conditions, this is less than 1% of total NIH funding (http://report.nih.gov/rcdc/categories/) and is primarily focused on preterm birth at around US$1,200 per case compared to US$18,000 per case for breast cancer and ovarian cancer. Yet the NIH allocates over US$1.9 billion to biodefense research. For low- and middle- income countries, which experience 98% of total neonatal deaths and a similar burden of stillbirths, the investment in research funding for neonatal survival is extremely low, perhaps around US$20 million per year, and the funds allocated to address intrapartum-related conditions are even lower. Defining specific funding allocations for research on intrapartum-related neonatal deaths is not possible in current research resource reporting, for either high- or low-income countries.

Given the large burden, the mismatch with investments and the short time frame before the MDG targets in 2015, evidence-based priority setting is imperative to accelerate progress in mortality reduction [20]. While there are strategies to reduce intrapartum-related neonatal deaths, the focus has been on having a functional health system to provide care at birth [21], with little consensus on how to strengthen weak systems over time [22], or how to address the 60 million annual home births [23],[24]. A recent series of papers involved a systematic review of evidence for interventions to reduce intrapartum-related deaths and screened almost 30,000 abstracts [8]. Several reviews summarized the evidence for obstetric care and for neonatal resuscitation [25],[26] and summarized health system actions that are needed [22],[27]. These reviews focused on the need for effective implementation strategies for intrapartum care in varying health system contexts and consistent measurement of pregnancy outcomes including maternal, neonatal and stillbirths. A previous survey of 173 policymakers and program managers reported on implementation gaps in programs to address intrapartum-related deaths [28]. The level of evidence for many intrapartum interventions is low and, while randomized trials for many accepted intrapartum care interventions may not be considered ethical, all recent reviews have highlighted the need for more investment in research [22]–[28]. As yet, no publication has set out a systematic research agenda on this topic.

## Priority Setting for Research Investments

The Child Health and Nutrition Research Initiative (CHNRI), linked with the Global Forum for Health Research, has summarized methodologies [29] developed over the last 20 years to set priorities for global health research investments (http://www.chnri.org/publications.php). Previous methods have included the Combined Approach Matrix [30] and the Delphi process [31]. These were the starting points for the development of the novel CHNRI approach to research priority setting, based on a well-defined context, transparent criteria, and independent input from investors, technical experts, and other stakeholders [31] (see Figure 2, right side). The CHNRI methodology has been proposed as a tool that could be used by those who develop research policy or invest in health research [32],[33]. The process examines (i) the full spectrum of research investment options, (ii) the potential risks and benefits that could result from investments in different research options, and (iii) the likelihood of achieving reductions of persisting burden of disease and disability through investments. CHNRI methodology has now been applied to a wide range of topics that include childhood pneumonia [34], diarrhea [35], neonatal infections [36], zinc supplementation [37], mental health [38], disability [39], primary health care [40], and also country-level priority setting in South Africa [41].

Several analyses have shown that around two-thirds of neonatal [42] and child deaths [43] could be prevented with existing interventions, but that there is a gap in current coverage, especially for the poorest families. The WHO's Child and Adolescent Health and Development Department (CAH) identified a need for a systematic approach to setting priorities for health research that could reduce this gap through health systems research while maximizing reductions in the five main causes of child death within the short time frame to the MDG 4 target. CAH recognized the potential usefulness of the CHNRI methodology, and in 2008 initiated a process to identify health research priorities to reduce mortality from the top five causes of child death, including intrapartum-related neonatal deaths. Several hundred technical experts from diverse backgrounds and all regions of the world took part in the exercise. In this paper we present the results and highest ranked research priorities to reduce mortality from intrapartum-related neonatal deaths by 2015.

## Methods

The CHNRI methodology for setting priorities in health research investments has four stages: defining the context and criteria for priority setting with input from investors and policymakers; listing and scoring research investment options by technical experts using predetermined criteria (Box 1); weighting the criteria according to wider societal values with input from other stakeholders; and computation and discussion of the scores and agreement between experts [32],[33],[44]. The CHNRI methodology, validity and potential limitations are detailed in Table S1.

**Box 1. Questions Answered by Technical Experts to Assign Intermediate Scores to Competing Research Options**

Possible answers: Yes = 1; No = 0; Informed but undecided answer: 0.5; Not sufficiently informed: blank.

***CRITERION 1:* Likelihood that research would lead to new knowledge (enabling a development/planning of an intervention) in ethical way**

1. Would you say the research question is well framed and endpoints are well defined?
2. Based on: (i) the level of existing research capacity in proposed research; and (ii) the size of the gap from current level of knowledge to the proposed endpoints; would you say that a study can be designed to answer the research question and to reach the proposed endpoints of the research?
3. Do you think that a study needed to answer the proposed research question would obtain ethical approval without major concerns?

***CRITERION 2:* Assessment of likelihood that the intervention resulting from proposed research would be effective**

1. Based on the best existing evidence and knowledge, would the intervention which would be developed/improved through proposed research be efficacious?
2. Based on the best existing evidence and knowledge, would the intervention which would be developed/improved through proposed research be effective?
3. If the answer to either of the previous two questions is positive, would you say that the evidence upon which these opinions are based is of high quality?

***CRITERION 3:* Assessment of deliverability, affordability, and sustainability of the intervention resulting from proposed research**

1. Taking into account the level of difficulty with intervention delivery from the perspective of the intervention itself (e.g., design, standardization, safety), the infrastructure required (e.g., human resources, health facilities, communication and transport infrastructure) and users of the intervention (e.g. need for change of attitudes or beliefs, supervision, existing demand), would you say that the endpoints of the research would be deliverable within the context of interest?
2. Taking into account the resources available to implement the intervention, would you say that the endpoints of the research would be affordable within the context of interest?
3. Taking into account government capacity and partnership requirements (e.g., adequacy of government regulation, monitoring and enforcement; governmental intersectoral coordination, partnership with civil society and external donor agencies; favorable political climate to achieve high coverage), would you say that the endpoints of the research would be sustainable within the context of interest?

***CRITERION 4:* Assessment of maximum potential of disease burden reduction**

As this dimension is considered "independent" of the others, in order to score competing options fairly, their maximum potential to reduce disease burden should be assessed as potential impact fraction under an ideal scenario, i.e., when the exposure to targeted disease risk is decreased to 0% or coverage of proposed intervention is increased to 100% (regardless of how realistic that scenario is at the moment—that aspect will be captured by other dimensions of priority setting process, such as deliverability, affordability and sustainability).

Non-existing interventions*

Maximum potential to reduce disease burden should be computed as "potential impact fraction” for each proposed research avenue, using the equation: PIF = [S_(i = 1 to n)_ P_i_ (RR_i_-1)]/[S_(i = 1 to n)_ P_i_ (RR_i_-1)+1];

where PIF is “potential impact fraction” to reduce disease burden through reducing risk exposure in the population from the present level to 0% or increasing coverage by an existing or new intervention from the present level to 100%; RR is the relative risk given exposure level (less than 1.0 for interventions, greater than 1.0 for risks), P is the population level of distribution of exposure, and n is the maximum exposure level.

Existing interventions**

Maximum potential to reduce disease burden should be assessed from the results of conducted intervention trials; if no such trials were undertaken, then it should be assessed as for non-existing interventions.

Then, the following questions should be answered:

1. Taking into account the results of conducted intervention trials**, or for the new interventions the proportion of avertable burden under an ideal scenario*, would you say that the successful reaching of research endpoints would have a capacity to remove 5% of disease burden or more?
2. To remove 10% of disease burden or more?
3. To remove 15% of disease burden or more?

***CRITERION 5:* Assessment of the impact of proposed health research on equity**

1. Does the present distribution of the disease burden affect mainly the underprivileged in the population?
2. Would you say that either (i) mainly the underprivileged, or (ii) all segments of the society equally, would be the most likely to benefit from the results of the proposed research after its implementation?
3. Would you say that the proposed research has the overall potential to improve equity in disease burden distribution in the long term (e.g., 10 years)?

### Stage 1: Define the Context and Criteria for Priority Setting

The aim of this particular exercise was to inform key global donors, investors in health research (especially of public funds), and international agencies about research investment policies that are expected to address MDG 4 in the most effective way. In choosing to focus on mortality, we nonetheless acknowledge the importance of non-fatal outcomes, such as the considerable burden of morbidity and sequelae related to intrapartum insults. In addition, while focusing on one condition (intrapartum-related neonatal deaths), there would be expected beneficial effects of investments from such research on related outcomes such as maternal deaths and stillbirths, and perhaps on the function of health systems and primary health care [9]. Furthermore, by setting a relatively short time frame (2015), research requiring a longer lead time was less likely to be highly ranked.

### Stage 2: List and Score Research Options Using Predetermined Criteria

Individuals with a wide range of technical expertise and regional representation were identified by a core team and by WHO staff and sent a formal invitation to participate. A list of research questions was drafted by the core team expert group based on recent systematic reviews [22]–[27] and a previous survey of experts [28]. The research questions were organized using the framework shown in Table S2. The expert group then reviewed the questions, adding to and refining the list. The final questions were sent to each technical group member in an Excel (Microsoft Word 2007) format for scoring.

Based on CHNRI methodology (Figure 1), five scoring criteria were applied: (i) answerability in an ethical way; (ii) likelihood of effectiveness; (iii) likelihood of deliverability, affordability, and sustainability; (iv) maximum potential impact on burden reduction; and (v) predicted impact on equity. The experts made a judgment on each proposed research question by answering the questions presented in Box 1.

### Stage 3: Solicit Input From Societal Stakeholders to Weight the Criteria

The five criteria for scoring (answerability, efficacy and effectiveness, deliverability, disease burden reduction, and effect on equity) may be perceived to be of varying importance and the value given to each criterion may vary with the perspective of stakeholders. For example, parents who have experienced a neonatal death may rate mortality reduction higher than a research funder who may value answerability, or a health system planner who may be most concerned with deliverability. Hence, CHNRI undertook an exercise to poll a wide range of stakeholders and to weight the criteria based on values assigned by them, as described elsewhere [44]. The weights applied in this exercise are explained in detail in Table S1.

### Stage 4: Compute "Research Priority Scores" and Average Expert Agreement

Completed worksheets were returned to the group coordinator. The overall research priority score (RPS) was computed as the mean of the scores for the five criteria, weighted according to the input from the stakeholders (Table S1), according to the formula:
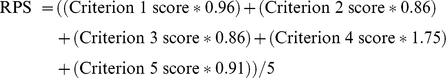



Average expert agreement (AEA) scores were also computed for each research question as the average proportion of scorers who agreed on the 15 questions asked. This was computed for each scored research investment option as:
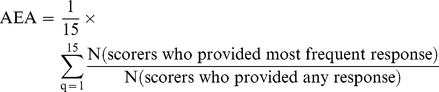
(where q is a question for which experts are being asked to evaluate competing research investment options, ranging from 1 to 15). For further details regarding the choice of methods, agreement statistics, and interpretation see Table S1.

## Results

Of the 26 experts who were approached and agreed to participate, 21 returned their scoring sheets within the allocated time, resulting in a completion rate of 81%. The scorers were evenly distributed across four regions (Africa [29%], Americas [29%], Asia/Middle East [19%] and Europe [24%]), and the regional distribution for non-responders was similar. Only 19% of responders (four) and non-responders (one) were female. Expertise covered clinical provision (midwifery, neonatology, obstetrics, pediatrics, and disability care), perinatal epidemiology, public health, and basic science, as well as both researchers and research funders. The full list of technical experts who were invited to participate, their expertise, and reasons for non-participation (for those who declined or failed to respond in time) are presented in Table S3.

The full list of 61 research options and the scores from each individual scorer are presented in Table S4. Questions are organized by delivery (health system research questions), development, discovery science, and epidemiology research themes. More questions were listed for delivery (28) than for development (11) or epidemiology (17), and far fewer for discovery (5). The scores ranged from 37 to 92 (potential 0 to 100), although almost all scores were over 50, suggesting that few of the research options were considered of little merit, and that the scoring system was able to help distinguish between a long list of mostly valuable options.

Table 1 shows the ten highest ranked questions after weighting. Of these, seven (70%) are related to delivery research, two to development research, one to epidemiological and none to discovery science questions. In the top ten ranked questions, the scores varied from 84 to 92. AEA varied from 0.42 to 0.79. Not surprisingly, the highest ranked research options tended to have a higher AEA. The lowest AEA, and also the lowest RPS, was for the question regarding amnioinfusion, suggesting that the question or the intervention may not have been well understood.

**Table 1:** The 15 research questions that achieved the highest overall research priority score (RPS), with average expert agreement (AEA) related to each question (total of 61 questions).

Rank	Proposed Research Question	Research Type	Answer-able?	Effec-tive?	Deliver-able?	Burden reduction?	Equitable?	AEA	RPS
1	Can community cadres of workers identify a limited number of high-risk conditions/danger signs (e.g., multiple pregnancy, breech, short maternal stature, etc.) and successfully refer women for facility birth? What is the predictive value and cost effectiveness?	Delivery	93	88	85	77	94	0.78	91.9
2	What strategies are effective in increasing demand for, and use of, skilled attendance (e.g., conditional cash transfers)?	Delivery	90	88	77	82	93	0.79	91.2
3	Behavioral/community participation package to improve recognition and acting for simplified danger signs for mother in labor, including transport and phone/radio communication ("emergency preparedness")?	Delivery	92	78	94	75	95	0.79	90.6
4	Effectiveness of community cadre roles, e.g., social support, bringing to facility when woman is in labor, danger recognition/referral?	Delivery	83	78	96	73	95	0.74	88.9
5	Does regular use of perinatal audit reduce the incidence of adverse outcomes related to acute intrapartum events?	Delivery	83	97	82	68	98	0.74	88.4
6	Can simpler/cheaper/more robust technology be developed for neonatal resuscitation (e.g., bag-and-mask, suction devices), and for resuscitation training (resuscitation dummies) and more feasible models of maintaining clinical competency for resuscitation?	Development	95	93	87	59	100	0.78	88.1
7	Does regular use of perinatal audit improve adherence to clinical standards for intrapartum care (e.g., use of partograph, monitoring of fetal heart rate, resuscitation etc.)?	Delivery	78	92	82	72	93	0.69	86.6
8	Can specific maternal complications (e.g., obstructed labor, hypertension, retained twin) with higher risk of intrapartum stillbirth, early neonatal death, or other unfavorable intrapartum-related outcomes be more simply predicted at an earlier stage?	Epidemiology	85	81	82	72	91	0.74	86.2
9	Can simpler clinical algorithms (recognition and management) be developed and validated for babies who require resuscitation at birth, and does this increase met need for resuscitation at birth?	Delivery	93	81	93	53	100	0.79	84.4
10	Can low-cost, robust, simple fetal heart monitors be developed and tested that are more user friendly than the Pinard—e.g., adaptations of Doppler FHM? Does use of such a device improve fetal heart rate monitoring and reduce intrapartum stillbirths and asphyxia-related outcomes?	Development	94	86	69	64	93	0.75	83.7

Two of the top four questions relate to how to most effectively generate demand for facility care at birth with specific mechanisms such as transport and communication schemes, or financial incentives and conditional cash transfers. The other two relate to use of community cadres and the roles that they might effectively play; for example, in screening for complications or for supportive transfer to facilities and companionship at birth.

Table 2 shows the ten lowest ranked questions after weighting, with a range of RPS scores (37–58) and AEA scores (0.42–0.64). Most of the five discovery research questions are among the lower ranks, being placed at 59, 55, 54, 39, and 25, respectively. Only two of the 11 development questions were in the lowest ten. However, the lowest-ranked question related to development/adaptation of amnioinfusion, which was ranked very low for burden effect and also for effectiveness. Of the 17 epidemiology questions, three fell in the lowest ten ranks and two related to early identification of infants with developmental delay after neonatal encephalopathy, with extremely low scoring for mortality reduction. Only two delivery research questions were in the lowest ten ranks, the lowest of which was about operationalizing care for diabetes in pregnancy in weak health systems (rank 58); this question scored low for burden reduction as well as for deliverability.

**Table 2:** The 15 research questions that achieved the lowest overall research priority score (RPS), with average expert agreement (AEA) related to each question (total of 61 questions).

Rank	Proposed Research Question	Research Type	Answerable?	Effective?	Deliverable?	Burden reduction?	Equitable?	AEA	RPS
52	What is the magnitude of misclassification between fresh stillbirths and early neonatal deaths, and which factors affect this misclassification? What decision rules (applicable in the community and hospital settings) can be used to differentiate?	Epidemiology	77	72	58	18	67	0.57	55.8
53	What is the positive and negative predictive value of a very low (<3) and a moderately low (4–6) Apgar score for neonatal encephalopathy (NE), death, etc.	Epidemiology	85	57	47	12	77	0.61	52.4
54	Can new, simple to use, robust technology be developed to better detect neonatal fetal distress or NE in low-income settings? e.g., amplitude-integrated EEG (cerebral function monitor, CFM) to identify NE for postnatal therapeutic interventions.	Development	75	62	23	26	79	0.66	52.4
55	What are the longer term outcomes of NE (6 months, 1 y, 5 y, and school function at 10 y), and is there an increased risk of death as well as disability and reduced school performance?	Epidemiology	79	81	32	11	74	0.64	51.8
56	Would novel micronutrient approaches reduce cerebral damage after insult (magnesium, nitrates, combinations etc.)?	Discovery	72	60	61	24	48	0.49	51.6
57	Does early identification of babies with development problems following NE improve utilization of services (feeding, physiotherapy, speech and language, hearing) and/or outcomes (hearing, vision, school performance)?	Delivery	83	43	47	4	78	0.60	46.9
58	Can care of diabetes in pregnancy be operationalized in context of weak health systems to reduce the risk of large for gest age babies?	Delivery	71	44	35	25	59	0.53	46.9
59	Would other novel drug treatments reduce cerebral damage after insult (allopurinol, epo, opioids, etc.)?	Discovery	70	60	42	10	37	0.51	41.7
60	Are there genes or other biomarkers that predict susceptibility to intrapartum hypoxic injury?	Discovery	50	62	10	18	48	0.60	37.0
61	Can the procedure of amnioinfusion be adapted to lower resource settings and would this impact asphyxia-related outcomes? Are there clinically important risks from the procedure?	Development	50	50	27	10	52	0.42	36.0

Table 3 shows the highest ranked questions for each of the four different research categories (description, discovery, development, and delivery). The epidemiology questions with the highest ranks (8, 11, and 19), were all questions with obvious clinical implications—for example, early prediction of intrapartum complications, risk of neonatal encephalopathy, or the need for resuscitation. The highest ranked discovery question (25th) related to the interaction of intrapartum infection/pyrexia and hypoxic injury. Several development research options were ranked highly (6, 10, and 16), and related to innovative technology for neonatal resuscitation, for detection of fetal distress and to approaches to maintaining provider competence for skills.

**Table 3:** Top three research questions within each instrument of health research: description (epidemiology), discovery (basic research), development (translational research), and delivery (operations research).

Description (Epidemiology)	Rank
1. Can specific maternal complications (e.g., obstructed labor, hypertension, retained twin) with a higher risk of intrapartum stillbirth, early neonatal death, or other unfavorable asphyxia-related outcome be more simply detected at an earlier stage?	8
2. What are the maternal and antenatal/intrapartum care risk factors for NE graded for mild, moderate, and severe in various settings?	11
3. What is the prevalence of babies requiring resuscitation in various settings? What is the prevalence for preterm and term babies?	19

Discovery (Basic Research)	
1. Can the synergy of infections/maternal pyrexia with neonatal encephalopathy (NE) be addressed through interventions e.g., antibiotic therapy for pyrexial women in labor?	21
2. Can new, simple to use, robust technology be developed to better detect fetal distress and NE in low-income settings? e.g., amplitude-integrated EEG (CFM) to identify NE for postnatal therapeutic interventions.	54
3. Would novel micronutrient approaches reduce cerebral damage after insult (magnesium, nitrates, combinations, etc.)?	56

Development	
1. Can simpler/cheaper/more robust technology be developed for neonatal resuscitation, (e.g., bag-and-mask, suction devices) and for resuscitation training (resuscitation dummies) and more feasible models of maintaining clinical competency for resuscitation?	6
2. Can low-cost, robust, simple fetal heart monitors be developed and tested that are more user friendly than the Pinard—e.g., adaptations of doppler FHM? Does use of such a device improve fetal heart rate monitoring and reduce intrapartum stillbirths and asphyxia-related outcomes?	10
3. Does distance learning for intrapartum care improve competencies? How do skills and cost compare with conventional training? How often does provider re-training need to be performed to maintain competency in neonatal resuscitation, for different cadres?	16

Delivery	
1. Can community cadres of workers identify a limited number of high-risk conditions/danger signs (e.g., multiple pregnancy, breech, short maternal stature, etc.) and successfully refer women for facility birth? What is the predictive value and cost effectiveness?	1
2. What strategies are effective in increasing demand for (and use of) skilled attendance? e.g., conditional cash transfers, etc.	2
3. Behavioral/community participation package to improve recognition and acting on simplified danger signs for mother in labor, including transport and phone/radio communication ("emergency preparedness").	3

## Discussion

As far as we know, this is the first systematically ranked research priority list for addressing the burden of almost 1 million intrapartum-related neonatal deaths, mostly occurring in the world's poorest families and in settings with too few frontline health workers. Three-quarters of the top ten priorities, and most of the top one-third of 61 research investment options, were dominated by delivery research (implementation). This is not surprising given the large number of preventable deaths with known solutions and the short time frame to give results in order to contribute to achievement of MDG 4 in 2015. The greatest immediate mortality gains could be achieved through better implementation of existing interventions, and greater investment in implementation research is an urgent need. The high-priority research questions identified in this exercise also have high scores for improving equity given the marked inequity in current coverage data regarding care at birth [1],[8],[22].

Given 60 million home births each year, it is appropriate that the top four priorities relate to closing the gap in skilled attendance at the time of birth for women and their babies, mainly by trying to bring them into facilities for birth through “pull” approaches (conditional cash transfers) or better linkages such as transport and communications, and to revisiting evidence-based, selective approaches to identifying pregnancies at greatest risk. Other themes in the “top ten” include improving facility based care with strategies such as audit (ranked 5, 7), and innovations for low-cost, simpler technology (ranks 6, 10), in addition to more questions regarding roles for community cadres (ranks 8 and 9).The scores for the top ten ranked options were close and it is possible that with a larger group of experts the rank orders would differ.

However, whilst delivery research investment is most likely to result in burden reduction in the shorter term, development and discovery research remain essential to develop new interventions to feed the delivery research pipeline [18]. The highest ranked question from the discovery research options was only at 25 out of 61. The ten lowest ranked options included the other four of the five initial discovery research options. This may reflect a systematic bias introduced by the specified context of a very short time frame (5 years). Discovery research often takes longer to be translated into measurable benefits in terms of mortality burden reduction, and by definition the link to reduction in mortality and inequity is less direct. The highest ranked discovery question related to the interaction of hypoxia and infection, which is particularly relevant in high burden settings where the prevalence of both conditions is high. Initial, small studies of head cooling for neonatal encephalopathy in high burden settings raise the question of whether infection may be a factor in the possible increased risk observed with cooling [45].

The development and epidemiological research questions mainly fell in the middle band. The highest ranked development option refers to simpler, cheaper, more robust technology for neonatal resuscitation, which is clearly critical given the major unmet need [26]. The highest ranked epidemiology question also echoed the need to revisit the radical move away from risk screening, asking if specific maternal complications (e.g., obstructed labor, hypertension, retained twin) with higher risk of intrapartum stillbirth, early neonatal death, or other unfavorable asphyxia-related outcome could be more simply detected at an earlier stage.

Although the CHNRI methodology represents a systematic attempt to deal with many of the challenges inherent in the complex process of research investment priority setting, there are still possible biases [29]. The initial list of questions is critical—if a given research investment option is not included, it cannot be scored and drops from view. Another important possible source of bias arises from the selection and response of the expert technical group. A larger scoring group and deliberate attempts to widen regional and professional variation appear to help reduce the risk of bias; in addition, due to independent scoring of lists, the ranking is less likely to be dominated by eloquent individuals, as may happen in traditional group discussion approaches to research priority setting. Limitations of CHNRI methodology and validation exercises are described and discussed in greater detail in Table S1.

## Conclusions

A strong political commitment has been made to MDG 4 and 5, but this commitment requires systematic changes in health research investment. Current investments mainly target the diseases prevalent in high-income countries and tend to favor basic research. This exercise highlights the research investments most likely to result in rapid progress towards MDG 4 in the countries with the most deaths. These primarily address delivery research and development research, particularly to increase the reach of some high impact interventions for the poorest and most heavily affected families. Competing research questions may all contribute to MDG 4 and certainly for the longer term agenda more investment is also required in discovery science. A more systematic approach with strategic investment in different instruments of health research would be expected to accelerate progress towards mortality reduction. While newborn survival has gained rapid attention in recent years, attention has yet to connect to adequate action [46]. Further progress in reducing deaths will depend on systematically addressing implementation and knowledge gaps, and targeted innovation where most of the deaths occur.

We challenge the research community, research funding organizations, and national research organizations to systematically address at least the top ten ranked research questions before 2015. These research options have the potential to prevent almost 1 million unnecessary neonatal deaths that occur every year, and also reduce an additional one million intrapartum stillbirths and the closely associated 350,000 maternal deaths [47].

## Supporting Information

Table S1 The CHNRI methodology for setting priorities in health research investments.
(0.03 MB PDF)

Table S2 CHNRI's starting framework from which a listing of many research options (level of 3–5-year research program) and research questions (level of individual research papers) were being proposed by technical experts to develop a consolidated list of research questions.
(0.02 MB PDF)

Table S3 Composition of the group of technical experts.
(0.03 MB PDF)

Table S4 Research options scored (61) and example of CHNRI scoring sheet.
(0.11 MB XLS)

## Abbreviations

AEA: average expert agreement
CAH: World Health Organization Child and Adolescent Health and Development Department
CHNRI: Child Health Nutrition Research Initiative
MDG: Millennium Development Goal
NE: neonatal encephalopathy
NIH: US National Institutes of Health
RPS: research priority score
World Health Organization
