# Supplementary material for: Setting Research Priorities to Reduce Almost One Million Deaths from Birth Asphyxia by 2015
Source: PLoS Med. 2011 Jan 11;8(1):e1000389. doi: 10.1371/journal.pmed.1000389 (PMC3019109; doi:10.1371/journal.pmed.1000389)
Supplement: Table S2 — CHNRI's starting framework from which a listing of many research options (level of 3–5-year research program) and research questions (level of individual research papers) were being proposed by technical experts to develop a consolidated list of research questions. (0.02 MB PDF) [file pmed.1000389.s002.pdf]

Table S2: CHNRI's starting framework from which listing of many research options (level of 3-5-year research program) and research questions (level of individual research papers) were being proposed by technical experts to develop a consolidated list of research questions.

RESEARCH INSTRUMENT	RESEARCH AVENUE	RESEARCH OPTION	RESEARCH QUESTION
Epidemiological research	Measuring the burden	(Technical experts were invited to use categorization of research avenues and instruments to systematically propose a number of 'research options' within each of the avenues; 'research options' correspond to the level of 3-to-5-year research program)	(Technical experts were invited to propose a number of very specific 'research questions', corresponding to the title of individual research papers, within each of the 'research avenues; eventually, after consolidation and removing of duplicate ideas, 154 such questions were retained for scoring)
	Understanding risk factors		
	Evaluating the existing interventions		
Health policy and systems research ("DELIVERY")	Studying capacity to reduce exposure to proven health risks		
	Studying capacity to deliver efficacious interventions		
Research to improve existing interventions ("DEVELOPMENT)	Research to improve deliverability		
	Research to improve affordability		
	Research to improve sustainability		
Research for development of new interventions ("DISCOVERY")	Basic research		
	Clinical research		
	Public health research		
