# Supplementary material for: Setting Research Priorities to Reduce Almost One Million Deaths from Birth Asphyxia by 2015
Source: PLoS Med. 2011 Jan 11;8(1):e1000389. doi: 10.1371/journal.pmed.1000389 (PMC3019109; doi:10.1371/journal.pmed.1000389)
Supplement: Table S3 — Composition of the group of technical experts. (0.03 MB PDF) [file pmed.1000389.s003.pdf]

Table S3. Composition of the group of technical experts

An overview of expert selection, participation and responses. All participation in this particular CHNRI exercise was voluntary and carried out without specific funding support. All the experts who were invited to participate in that exercise had a track record of research on "birth asphyxia", either as clinicians, epidemiologists, social scientists, public health specialists or funders.

**Scorers**			
	**Name**	**Expertise**	**Affiliation**
1	Joy E Lawn	Clinician, perinatal epidemiologist, public health expert, researcher	Non governmental organization, academic
2	Zulfi Bhutta	Clinician, public health expert, researcher	Academic, Ministry of Health advisor
3	Gary L Darmstadt	Clinician, public health expert, researcher, donor	Foundation, Research funder
4	Lyn Sibley	Midwife, public health expert, researcher	Academic
5	Vinod Paul	Neonatologist, public health expert, researcher	Academic, Ministry of Health advisor
6	Matthew Ellis	Disability expert, pediatrician, public health expert	Academic and Gov service provider
7	David Osrin	Clinician, perinatal epidemiologist, public health expert, researcher	Academic
8	John Wyatt	Neonatologist, ethical expert, basic science, researcher	Academic
9	Jennifer Kurinczuk	Clinician, perinatal epidemiologist, public health expert, researcher	Academic, government public health service
10	Bob Pattinson	Obstetrician, public health expert, researcher	Academic
11	Justus Hofmeyr	Obstetrician, public health expert, researcher	Academic and Gov service provider
12	Ola Saugstad	Clinician, basic scientist, researcher, basic science, researcher	Academic
13	Nalini Singhal	Neonatologist, public health expert,	Academic and Gov service provider
14	Dave Woods	Neonatologist, health training expert	Academic and Gov service provider
15	Siddarth Ramji	Neonatologist, public health expert, researcher	Academic and Gov service provider, Ministry of health Advisor
16	Steve Wall	Neonatologist, public health expert	Non governmental organization,
17	CC Lee	Pediatrician, public health expert, researcher	Academic and service provider
18	Staffan Bergstrom	Obstetrician, public health expert, researcher	Academic
19	Mohamed Mohamed	Clinician, public health expert	Academic and service provider
20	Mike English	Clinician, public health expert, researcher	Academic, Ministry of Health advisor
21	Mario Merialdi	Obstetrician, perinatal epidemiologist, public health expert	United Nations staff member (WHO)
**Invited but did not score**			
	**Name**	**Expertise**	**Affiliation**
	Matthews Mathai	Obstetrician, public health expert	Had already participated in a similar pilot exercise in early phase of CHNRI methodology development
23	Simon	Statistician, public health expert	Had already participated in a

	Cousens		similar pilot exercise in early phase of CHNRI methodology development
24	Vyta Senikas	Obstetrician, public health expert	On sabbatical
25	Joseph de Graft Johnson	Clinician, public health expert	Extensive travel for work
26	Vincent Fauveau	Obstetrician, public health expert	No reason given
	Shoo Lee	Neonatologist	Email address out of date and never established contact to enable invitation
